# A Treatise on Epidemic Cholera

**Published:** 1855-10

**Authors:** 


ART. VII.—*A Treatise on Epidemic Cholera.* By HORATIO GATES JAMESON, Senr., M. D., Member of the Medical and Chirurgical Faculty of Maryland; Professor of Surgery; Member of Philosophical Societies of Berlin and Moscow. Philadelphia: Lindsay & Blakiston. 1855.

By a sad coincidence the functions of the reviewer are in this case merged with those of the writer of obituaries. Dr. Jameson, the author of the volume before us, died in New York, on the 26th of August last, at the age of

76. Dr. J. was then engaged in procuring subscriptions for his forthcoming work.

Were the harsher duties of the critic required in this case, we should forego them. But Dr. J. was no young man rushing unprepared into the field of authorship, and through the present volume, like all others on this peculiar subject, is unsatisfactory, it embraces a large and valuable mass of facts of much importance to the practitioner.

Dr. J. contends that cholera is the product of a new modification of electricity. We do not find that he affords any definite proof either that there is any such new modification, or that, existing, it is the cause of cholera; but whatever may be the merits of this question, we concur fully in the succeeding postulate which in no manner involves the action of electricity. Dr. J. says that "we may reasonably ascribe the course of the malady to a gradual impairment of the nervous power—originating in the splanchnic system." Such is our belief; but whether upon it can be based any mode of treatment, more promising than that by opium, is still a question.

We have in this number no space for any satisfactory consideration of this work. Although deficient in arrangement, and occasionally giving evidence of the great age of the writer, it has the merit of resting its conclusions upon a long experience and a close observation, and will be found valuable for reference in those visitations which city practitioners must now almost yearly expect and dread.

